# Visual IoT Security: Data Hiding in AMBTC Images Using Block-Wise Embedding Strategy †

**DOI:** 10.3390/s19091974

**Published:** 2019-04-27

**Authors:** Yu-Hsiu Lin, Chih-Hsien Hsia, Bo-Yan Chen, Yung-Yao Chen

**Affiliations:** 1 Dept. Electrical Engineering, Allied AI Biomedical Research Center, Southern Taiwan University of Science and Technology, Tainan 710, Taiwan; yhlin1108@stust.edu.tw; 2 Dept. Computer Science and Information Engineering, National Ilan University, Ilan 260, Taiwan; chhsia625@gmail.com; 3 Graduate Inst. Automation Technology, National Taipei University of Technology, Taipei 106, Taiwan; t105618004@ntut.edu.tw

**Keywords:** Visual Internet of Things, data hiding, visual sensing data security, secure image transmission, absolute moment block truncation coding (AMBTC)

## Abstract

This study investigates combining the property of human vision system and a 2-phase data hiding strategy to improve the visual quality of data-embedded compressed images. The visual Internet of Things (IoT) is indispensable in smart cities, where different sources of visual data are collected for more efficient management. With the transmission through the public network, security issue becomes critical. Moreover, for the sake of increasing transmission efficiency, image compression is widely used. In order to respond to both needs, we present a novel data hiding scheme for image compression with Absolute Moment Block Truncation Coding (AMBTC). Embedding secure data in digital images has broad security uses, e.g., image authentication, prevention of forgery attacks, and intellectual property protection. The proposed method embeds data into an AMBTC block by two phases. In the intra-block embedding phase, a hidden function is proposed, where the five AMBTC parameters are extracted and manipulated to embed the secret data. In the inter-block embedding phase, the relevance of high mean and low mean values between adjacent blocks are exploited to embed additional secret data in a reversible way. Between these two embedding phases, a halftoning scheme called direct binary search is integrated to efficiently improve the image quality without changing the fixed parameters. The modulo operator is used for data extraction. The advantages of this study contain two aspects. First, data hiding is an essential area of research for increasing the IoT security. Second, hiding in compressed images instead of original images can improve the network transmission efficiency. The experimental results demonstrate the effectiveness and superiority of the proposed method.

## 1. Introduction

Rapid technological advances in network technology have made it possible to access large amounts of information on the Internet. However, the Internet environment is not secure enough for personal information to be stored safely. To ensure that personal information is not easily stolen or distributed maliciously, data hiding schemes are useful techniques for the Internet of Things (IoT) and visual IoT [1]. Due to the security problems of many image encryption schemes summarized in [2], data hiding is a critical area of research for increasing the security of the network information. 

Nowadays, the data hiding technique has been investigated and published by many researchers. Data hiding is of two main kinds: reversible and irreversible. Reversible data hiding methods [3,4,5,6] emphasize that the original image can be recovered after data hiding. Thus, the images that require high detail information use reversible data hiding, such as mechanical drawing and medical imaging. However, to maintain the reversibility of the stego (i.e., the data-embedded) image, the amount of data that can be embedded will decrease or additional information would be required to recover from the stego image. 

**Figure 1:**
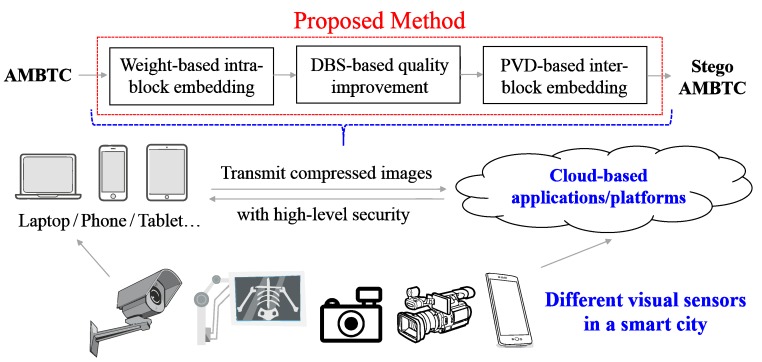
Conceptual framework and the application scenario which illustrate the usage of the proposed method. In a smart city, the proposed method can perform data-hiding and compression jointly to increase the security level of visual sensing data.

For irreversible data hiding [7,8], the least significant bit (LSB), exploiting modification directions (EMDs) and pixel value differencing (PVD) might be three of the most common techniques. The LSB method embeds binary secret data in the host image by replacing the LSB of the pixels to generate the stego image [9]. LSB is used because it can keep the minimal modification of the pixels while embedding data in the image. The EMD method embeds the (2n+1)−ary digit data in *n* stego pixels, which at the most adjusts one of the stego pixels by increasing or decreasing its value by 1 (i.e., ±1) and leaving the rest unchanged [10]. The key advantage of EMD is that it decreases the number of pixels that need to be adjusted for data embedding; thus, it improves the quality of the stego image. The PVD method embeds the fixed size of data in the host image by adjusting the difference between the two pixels in pairs [11]. To reduce the modification between the two pixels in a pair that substantially decreases the quality of the stego image, the PVD method usually divides the pixel values into several sections of the same size. Although the irreversible data hiding technique cannot recover the stego image back to what it was, it usually does not need additional data for recovery and data extraction. In addition, the irreversible data hiding technique can achieve a higher hiding capacity. Therefore, in this paper, we propose the irreversible scheme. Figure 1 illustrates the application scenario (and the overall framework) of the proposed method.

Using the image data hiding techniques, we can embed secret data into an image to protect the owner’s intellectual property and prevent abuse or tampering. Image data hiding is typically based on some specific image compression format [12,13,14,15,16]. When the image is compressed, it requires less memory for storage and is more efficient for transmission via the Internet. Image compression formats can be divided into lossy image compression and lossless image compression. Although lossy compression cannot recover the original image, it significantly reduces the image size, i.e., the compression rate performance of lossy compression is better than that of lossless compression. In this study, we use a lossy image compression method called absolute moment block truncation coding (AMBTC), to effectively compress the image. 

The advantage of the AMBTC compression technique is that it requires quite low computation power and can still achieve an acceptable image quality. Therefore, it is suitable for low-energy devices. Recently, several researchers have investigated the AMBTC-based data hiding methods. Ou and Sun [17] proposed a steganography method that classifies the AMBTC blocks into smooth or complex blocks. In [17], two embedding schemes are designed according to the block type, which helps the authors to exploit the different block properties more efficiently. Tang et al. [18] proposed an adaptive image steganography method that uses the interpolation technique to improve the hiding performance. Lin et al. [19] proposed an image authentication method that also divides the AMBTC blocks into smooth or complex blocks. In smooth blocks, the authentication code is embedded into the bitmap, whereas in complex blocks, the authentication code is embedded to adjust the quantization levels using a reference table. Huang et al. [20] proposed a hybrid secret hiding scheme for data hiding in AMBTC images, in which, similar to [17] and [19], two types of blocks are classified. Then, data embedding in the complex blocks exploits the difference between high and low mean values of a block; therefore, it achieves a higher embedding capacity than the previous methods. Al-Salhi and Lu [21] presented an image concealment scheme that uses PVD techniques. Furthermore, the human visual system and adaptive neural networks are integrated to improve the image quality. Hong et al. [22] proposed an AMBTC-based data hiding method that uses a suppress threshold mechanism and a perturbation technique to minimize the block distortion in the stego image.

However, image quality image is inevitably degraded or a few image details are lost after compression by AMBTC and the data hiding procedure. Thus, this study adopts a halftone image optimization technology called Direct Binary Search (DBS). DBS is widely used instead of the heuristic approach to optimize halftone images by using a human visual system to minimize the total squared perceived error between a continuous tone image and a halftone image [23,24]. In this study, we adopt the Swap operator from the DBS framework and use this operator to optimize the stego image that is compressed and contains the hidden data. Using the Swap operator not only makes the stego image visually closer to the original image without affecting the hiding data, but also upgrades the image quality. 

## 2. Related Work (AMBTC Compression)

As mentioned, AMBTC is a lossy image compression method which requires quite low computation power [25]. Therefore, AMBTC is suitable for real-time embedded system applications and can obtain a satisfactory compressed image quality. In AMBTC, the input image is first divided into non-overlapped square blocks with the side length of *n* (i.e., the block size *BS* equals to *n*). To compress the image, the average value of all the pixel values in each block is calculated by:

(1) $$\bar{x}=\frac{1}{n^{2}}\sum_{i=1}^{n\times n}x_{i},$$

where $x_{i}$ indicates the *i*-th pixel value of the block. 

The average pixel value is then used as a threshold to generate a n×n bit map by comparing $x_{i}$ with the pixel values in a block. For the case that $x_{i}$ is larger than the pixel value, the bit map value is set as 0 (later, it is replaced by the low mean value in the AMBTC compressed block) at the position; otherwise, it is set as 1 (replaced by the high mean value in the AMBTC compressed block). After comparison, the number of pixels whose pixel value is less than $x_{i}$ is referred as the number of low-mean bits (*NL*), and the number of pixels whose pixel value is larger than or equal to $x_{i}$ is referred as the number of high-mean bits (*NH*). For AMBTC compression, low mean and high mean are the two quantization levels calculated using the following:

(2) $$LM=\frac{1}{NL}\sum_{x_{i}<\bar{x}}x_{i},$$

and

(3) $$HM=\frac{1}{NH}\sum_{x_{i}\geq \bar{x}}x_{i}.$$

**Figure 2:**
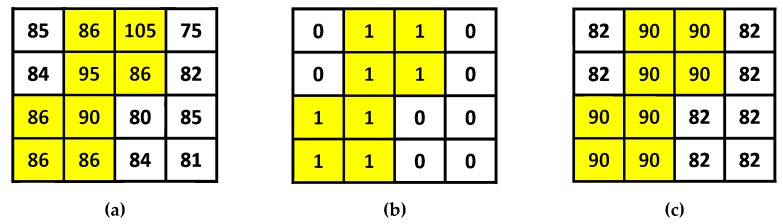
Illustration of the Absolute Moment Block Truncation Coding (AMBTC) compression. (**a**) Original image block, where the average pixel value $\bar{x}$ = 86 using (1). (**b**) The bit map used in AMBTC, where *NH* = 8 and *NL* = 8. (**c**) The AMBTC compressed block, where *BS* = 4, *HM* = 90, and *LM* = 82.

Figure 2 shows an example of the AMBTC compression. Different from the original grayscale image block, in an AMBTC block, the pixel values are only two quantization levels (low mean and high mean), and thus it can save the memory for storage. In addition, the first absolute moment of each original image block is maintained.

## 3. Proposed 2-Phase Intra- and Inter-Block Embedding Method

### 3.1. Intra-Block Embedding Phase

This study presents a 2-phase (intra-block and inter-block) data hiding strategy by manipulating AMBTC parameters. In the intra-block embedding phase, the hidden data are embedded into an AMBTC block by proper modification of the set containing five AMBTC parameters:

(4) θ={HM, LM, NH, NL, BS},

where *HM*, *LM*, *NH*, *NL*, and *BS* indicate, respectively, high mean, low mean, number of high-mean bits, number of low-mean bits, and AMBTC block size. The hidden function is defined as:

(5) H(θ)=(HM×1)+(LM×2)+(NH×3)+(NL×4)+(BS×5).

The secret bits to be embedded are converted to their decimal representation S. For example, $S={\left(11010\right)}_{2}=26$ in the case of 5-bit secret data. The goal of intra-block embedding is to adjust the parameters in θ so that S is equal to the result obtained from the modulo operation:

(6) $$S=\mathrm{mod}(H,2^{n})$$

where *n* is the size of the secret bits embedded in a hidden function. Because the n-bit secret data are embedded by utilizing the modulo operation of H with the divisor $2^{n}$, the simplest case occurs when $\mathrm{mod}(H,2^{n})$ is equal to S (no adjustment is required). Otherwise, the difference between $\mathrm{mod}(H,2^{n})$ and S must be eliminated by determining the embedding difference d such that $S-\mathrm{mod}(H+d,2^{n})=0$. Two directions can be used to adjust the embedding difference:

(7a) $$d_{1}=S-\mathrm{mod}(H,2^{n}),$$

(7b) $$d_{2}=\{\begin{array}{c} d_{1}-2^{n},\;\mathrm{if}\;\mathrm{sgn}(d_{1})=1\;\\ 2^{n}-\left|d_{1}\right|,\;\mathrm{if}\;\mathrm{sgn}(d_{1})=-1 \end{array},$$

where *sgn* is the signum function. However, too many combinations of the parameters can lead to the result of (6) for the given secret data. It is difficult to find the optimal parameters efficiently.

To address this problem, the concept of nominal parameters is proposed. As shown in Table 1, three parameters (*NH*, *NL*, and *BS*) in θ are referred to as the “nominal parameters”, which means that adjusting these three parameters in (6) does not change the actual information of an AMBTC block. Specifically, their actual value is maintained; however, when the hidden function value is calculated, the values of the three parameters are nominally changed, as the form of the trio code is changed. For example, when *HM* = 120, *LM* = 80, *NH* = 9, *NL* = 7, and *BS* = 4, the hidden function value is:

(8) H(θ)=(120×1)+(80×2)+(9×3)+(7×4)+(4×5)=355

However, if the form of the trio code is changed to $\left(L_{i},H_{i},{\bar{B}}_{i}\right)$, the hidden function value becomes:

(9) $$H(\theta^{\prime })=(120\times 1)+(80\times 2)+(10\times 3)+(9\times 4)+(5\times 5)=371$$

Note that for embedding 5-bit secret data, which has 32 combinations, according to Table 1, if the bottom trio code is transmitted, the change in the resulting hidden function value designed to be 16 (i.e., 1×3+2×4+1×5), which is coincidentally half of 32. It will significantly reduce the search range and alleviate the effect of modifying θ to maintain image quality. Moreover, according to the rule listed in Table 1, two more directions can be used to adjust the embedding difference:

(10a) $$d_{3}=S-\mathrm{mod}(H+16,2^{n})$$

and

(10b) $$d_{4}=\{\begin{array}{c} d_{3}-2^{n},\;\mathrm{if}\;\mathrm{sgn}(d_{3})=1\;\\ 2^{n}-\left|d_{3}\right|,\;\mathrm{if}\;\mathrm{sgn}(d_{3})=-1 \end{array}.$$

This work utilizes a two-step search approach to adjust the parameters *HM* and *LM*. In step 1, the final embedding difference d is selected from $d_{1}\sim d_{4}$ if it has the minimum absolute value, such that:

(11) $$d=d_{i},\;\mathrm{if}\;\left|d_{i}\right|=\arg\underset{i}{\min}\left|d_{i}\right|.$$

In step 2, the parameters (HM,LM) are adjusted according to d, but the search constraints for the optimal *HM* (and *LM*) are set such that the variation of both must be less than a predefined threshold of six (an empirical value). Although using a larger threshold can expand the candidate pool of the eligible combinations, the quality of the stego image will degrade. By contrast, using a smaller threshold will limit the number of eligible combinations. For the combinations that pass the two-step search, the concept of mean square error (MSE) is used to determine the final parameters. The MSE value of a block can be expressed as follows:

(12) $$\mathrm{MSE}=\frac{1}{BS^{2}}\sum_{n=1}^{BS^{2}}{\left(I_{n}-P_{n}\right)}^{2},$$

where $I_{n}$ is the pixel value of the original image block, and $P_{n}$ is the pixel value of the stego block.

The following example describes the data hiding (and the data extraction) of intra-block embedding procedure. Assume that the original AMBTC parameters are θ={HM=120, LM=80, NH=7, NL=9, BS=4}, with H(θ)=357, and the 5-bit secret data is $S={\left(11010\right)}_{2}=26$. When (7) and (10) are applied, the four directions that can eliminate the difference between $\mathrm{mod}(H,2^{n})$ and S are $d_{1}=21,\;d_{2}=-11,\;d_{3}=5$, and $d_{4}=-27$. In addition, according to (11), the final embedding difference is d=5, in which the form of the trio code is $\left(L_{i},H_{i},{\bar{B}}_{i}\right)$. Six candidate pairs exist that increase the hidden function value by 5 and satisfy the search constraints:

(Candidate 1) $\left(HM^{\prime }=HM-5,\;LM^{\prime }=LM+5\right)$;
(Candidate 2) $\left(HM^{\prime }=HM-3,\;LM^{\prime }=LM+4\right)$;
(Candidate 3) $\left(HM^{\prime }=HM-1,\;LM^{\prime }=LM+3\right)$;
(Candidate 4) $\left(HM^{\prime }=HM+1,\;LM^{\prime }=LM+2\right)$;
(Candidate 5) $\left(HM^{\prime }=HM+3,\;LM^{\prime }=LM+1\right)$; and
(Candidate 6) $\left(HM^{\prime }=HM+5,\;LM^{\prime }=LM+0\right)$,

where the symbol “prime” indicates that the modification of parameters comes from the embedding of intra-block secret data. Finally, after the resulting MSE values of the six candidate pairs are compared, the candidate whose value corresponding with the minimum mean square error is selected. 

Assume that the above fourth candidate pair is selected after comparing among the MSE values, and the stego AMBTC parameters become θ={HM=121, LM=82, NH=7, NL=9, BS=4}. In addition, the trio code is $\left(L_{i},H_{i},{\bar{B}}_{i}\right)$. At the receiving end, according to Table 1, the hidden function value is recalculated as 378. Then, the 5-bit secret code ${\left(11010\right)}_{2}$ can be extracted by using (6) and applying the decimal-to-binary conversion.

### 3.2. Quality Improvement Phase

**Figure 3:**
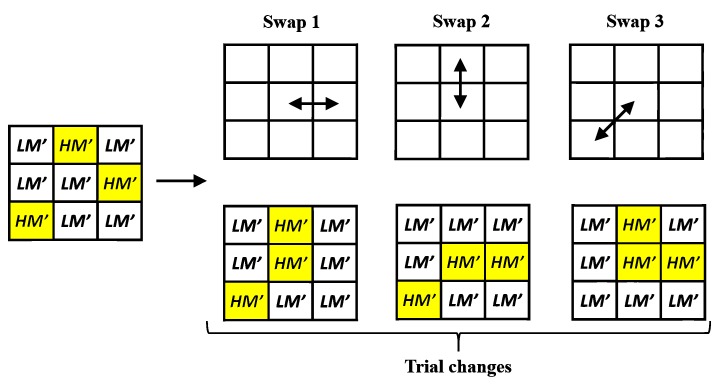
Illustration of the Direct Binary Search (DBS) swap operator, which is used in the quality improvement phase.

**Figure 4:**
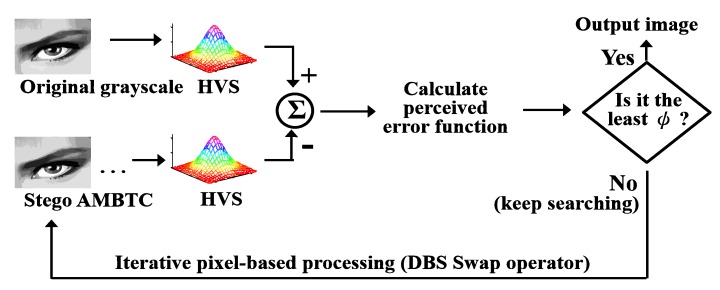
Concept of the DBS optimization framework used in this study.

In this subsection, we want to improve the quality of the stego image which comes from the intra-block embedding phase. Because the hidden data are embedded by adjusting the AMBTC parameters to certain fixed values that satisfies (6), the challenging part is: *How to improve the image quality of current stego image without changing the AMBTC parameters used in (6)?* To address this problem, this work applies a modified DBS optimization framework which only uses the swap operator. Using the DBS method enables us to take the human vision system (HVS) into account in the quality Improvement phase. Figure 3 shows the concept of DBS swap operator, and Figure 4 shows the concept of DBS method. 

In this study, the perceived grayscale image ($\tilde{g}$), the perceived stego AMBTC image ($\tilde{h}$) and the perceived error image ($\tilde{e}$) are defined, respectively, as

(13) $$\tilde{g}\left(x,y\right)=\sum_{m,n}g\left[m,n\right]P_{HVS}\left(x-mX,y-nY\right),$$

(14) $$\tilde{h}\left(x,y\right)=\sum_{m,n}h\left[m,n\right]P_{HVS}\left(x-mX,y-nY\right),$$

(15) $$\tilde{e}\left(x,y\right)=\sum_{m,n}\left(h\left[m,n\right]-g\left[m,n\right]\right)P_{HVS}\left(x-mX,y-nY\right),$$

where $P_{HVS}$ indicates the HVS filter which simulates the property of human eye, and (X,Y) indicates the lattice basis of addressable dot. The error image indicates the error image between the original grayscale image and the stego image. The perceived error function is defined as

(16) $$\phi ={\int_{x}\int_{y}\left|\tilde{e}\left(x,y\right)\right|}^{2}dxdy,$$

DBS [23] is a pixel-based processing method which changes the regional 3×3 pattern centered at the current processing position iteratively. For the DBS swap operator, it swaps the current mean value with the mean value of its eight nearest neighbors and calculate the effects of all the trial changes. If an effect of trial change leads to the smallest error function, the corresponding trial swap is accepted. Furthermore, the DBS method introduces an autocorrelation function and a cross-correlation function to accelerate the computational efficiency. Because swapping the mean values between two adjacent pixel positions does not change the number of high-mean (low-mean) bits, the hidden function value is maintained, and the embedded data are preserved. 

### 3.3. Reversible Inter-Block Embedding Phase

Inter-block embedding is performed on a pair of two adjacent blocks. First, all the AMBTC blocks B are ordered and divided into two sets: even-order blocks $B_{e}$ and odd-order blocks $B_{o}$:

(17) $$\left\{ \begin{array}{l} B_{e}(n)=B(2n)\\ B_{o}(n)=B(2n+1) \end{array}\right..$$

**Figure 5:**
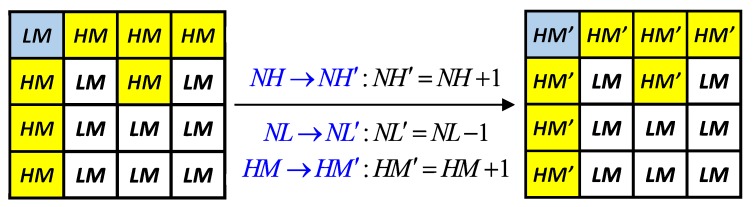
Illustration of compulsorily modifying parameters to satisfy search constraints and maintain hidden function values simultaneously.

For a pair of $\left(B_{e},B_{o}\right)$, the intra-block embedding of block $B_{o}$ is undertaken first. Subsequently, block $B_{e}$ is subject to the same intra-block embedding procedure; however, we add one more search constraint in the final step of determining (HM,LM), namely that both mean value differences between the two HM (and LM) values must be even numbers:

(18) $$\{\begin{array}{c} DH=HM_{e}-HM_{o}\;\mathrm{is}\;\mathrm{an}\;\mathrm{even}\;\mathrm{number}\\ DL=LM_{e}-LM_{o}\;\mathrm{is}\;\mathrm{an}\;\mathrm{even}\;\mathrm{number} \end{array},$$

where $HM_{e}$ ($LM_{e}$) and $HM_{o}$ ($LM_{o}$) represent the high (low) mean values from the even-order block and the odd-order block, respectively. Sometimes, no candidate parameter pairs of block $B_{e}$ are found because of the constraints used. In this case, we compulsorily change the values of using the method shown in Figure 5. Note that, for the example shown in Figure 5, the value of HM is increased by 1; however, the hidden function value is unchanged (i.e., the secret data of the intra-block embedding are not damaged) because the values of (NH,NL) are modified accordingly.

By setting the constraint of even parity in (18) in advance, two extra secret bits can be embedded between blocks $\left(B_{e},B_{o}\right)$ according to the rules in Table 2, where the symbol “double prime” indicates that the modification of parameters comes from the embedding of inter-block secret data.

In this work, we propose a reversible inter-block embedding scheme, meaning that, in the secret extraction phase, the change resulting from the interblock embedding procedure is reversible. Therefore, the quality of the stego image can be preserved to a certain extent. The following example describes the inter-block embedding and extraction procedures. Assume that the high (low) mean values of the block pair $\left(B_{e},B_{o}\right)$ are $\left(HM_{e},LM_{e})=(137,104\right)$ and $\left(HM_{o},LM_{o})=(119,80\right)$, and that the 2-bit secret code is ${\left(10\right)}_{2}$. As per Table 2, the modified parameters of $B_{e}$ become $\left(HM_{e}^{\prime \prime },LM_{e}^{\prime \prime })=(138,104\right)$, whereas in block $B_{o}$, $\left(HM_{o}^{\prime \prime },LM_{o}^{\prime \prime })=(HM_{o},LM_{o}\right)$ is maintained. At the receiving end, the differences between the mean values are first calculated as $DH^{\prime \prime }=HM_{e}^{\prime \prime }-HM_{o}^{\prime \prime }$ and $DL^{\prime \prime }=LM_{e}^{\prime \prime }-LM_{o}^{\prime \prime }$. The 2-bit secret code can be extracted by utilizing the modulo operation

(19) $$\mathrm{Code}\;1=\mathrm{mod}(DH^{\prime \prime },2),\;\mathrm{Code}\;2=\mathrm{mod}(DL^{\prime \prime },2).$$

Furthermore, the original mean values can be retrieved using

(20) $$\{\begin{array}{c} HM_{e}=HM_{e}^{\prime \prime }-\mathrm{mod}(DH^{\prime \prime },2)\\ LM_{e}=LM_{e}^{\prime \prime }-\mathrm{mod}(DL^{\prime \prime },2)\; \end{array}.$$

## 4. Experimental Results and Discussions

**Figure 6:**
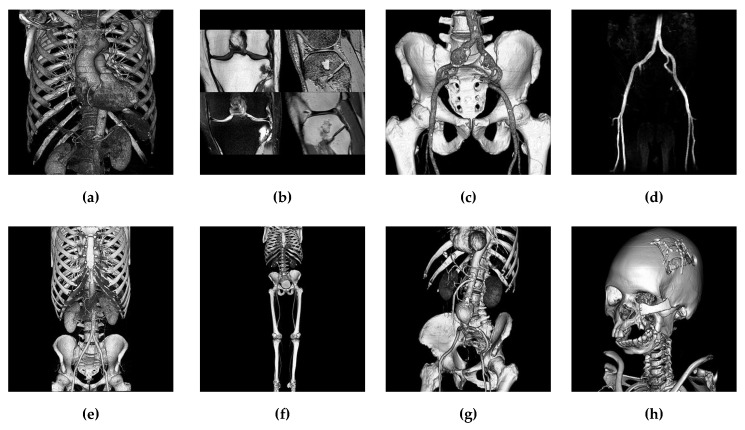
The eight test images. (**a**) Artifix. (**b**) Knee. (**c**) Macosessix. (**d**) Matrix. (**e**) Mecanix. (**f**) Obelix. (**g**) Panoramix. (**h**) Phenix.

For evaluation, the performance of the proposed method is compared with the recently published methods in [17] (2015), [20] (2017), and [22] (2017). Because medical images are essential sources of visual data in smart cities (for example, a smart home-based health care system which needs to transmit mass medical images for telemedicine), eight 400 × 400 test images are collected from the public database [25] (Figure 6). 

Moreover, for the methods in [17,20,22], blocks are classified as smooth and complex blocks. Similarly, in our comparison, the concept in [17] is adopted to embed data into smooth blocks, and the proposed method is used to embed data into complex blocks. In addition to comparing the payload (i.e., data capacity in units of bits), this work adopts two image quality measures to fairly evaluate the performance of each method. The first quality measure is called HVS–based Peak Signal-to-Noise Ratio (HPSNR), which integrates the characteristics of HVS low-pass filter with the traditional Peak Signal-to-Noise Ratio (HPSNR). The HPSNR value can be expressed as follows;

(21) $$\mathrm{HPSNR}=10\times \log\left(\frac{P\times Q\times 255^{2}}{\sum_{P,Q}{\left[\sum_{m,n}q_{m,n}(g_{i+m,j+n}-h_{i+m,j+n})\right]}^{2}}\right)\;(\mathrm{dB}),$$

where (P,Q) indicates the image size, q indicates the HVS low-pass filter; $g_{i,j}$ and $h_{i,j}$ indicate the pixels values of the input grayscale image and the resulting stego AMBTC image, respectively.

The second quality measure is called Mean Structural Similarly Index Measure (MSSIM), which is an averaging result of multiple number of the traditional Structural Similarly Index Measure (SSIM). The MSSIM value can be expressed as:

(22) $$\mathrm{MSSIM}=\sum_{j=1}^{M}\mathrm{SSIM}(g_{j},h_{j}).$$

where *M* is the number of SSIM local windows (set as 3 in this paper); $g_{j}$ and $h_{j}$ represent the image block within the *j*-th window, from the input grayscale image and the resulting stego AMBTC image, respectively. Table 3 shows the overall comparison of the four methods as the threshold value (for distinguishing smooth and complex blocks) is equal to 2. As shown in Table 3, because most AMBTC blocks are classified as complex blocks due to the small threshold, the quality of stego AMBTC image using the proposed method achieves very satisfactory results. As shown in Table 3, because most AMBTC blocks are classified as complex blocks due to the small threshold, the quality of stego AMBTC image using the proposed method achieves very satisfactory results. It demonstrates that the proposed quality improvement phase is effective.

When a larger threshold value is applied, more blocks are classified smooth blocks. For the test image Matrix, embedding capacity for different methods is similar because the number of smooth blocks is considerably larger than that of complex blocks. However, the payload rows of Table 3 and Table 4 show that the proposed method completely outperforms the other three methods in terms of embedding capacity. The results validate that the proposed intra- and interblock embedding schemes could utilize the property of the complex blocks to embed data. The comparison of the image quality reveals that the proposed method has higher HPSNR/MSSIM values than the methods in [17,20,22], which are also higher than those for some original AMBTC images. Furthermore, for the comparison of computational performance, the average processing times required to produce a stego image are 5.47 ms (in [17]), 6.11 ms (in [20]), 5.85 ms (in [22]), and 6.93 ms (in the proposed method). All the data hiding methods are implemented in the Windows 7 operating system, with 2.7 GHz CPU and 4 GB RAM. Although adding the modified DBS into the proposed data hiding method increases the processing time, the improvement of the image quality is remarkable.

**Figure 7:**
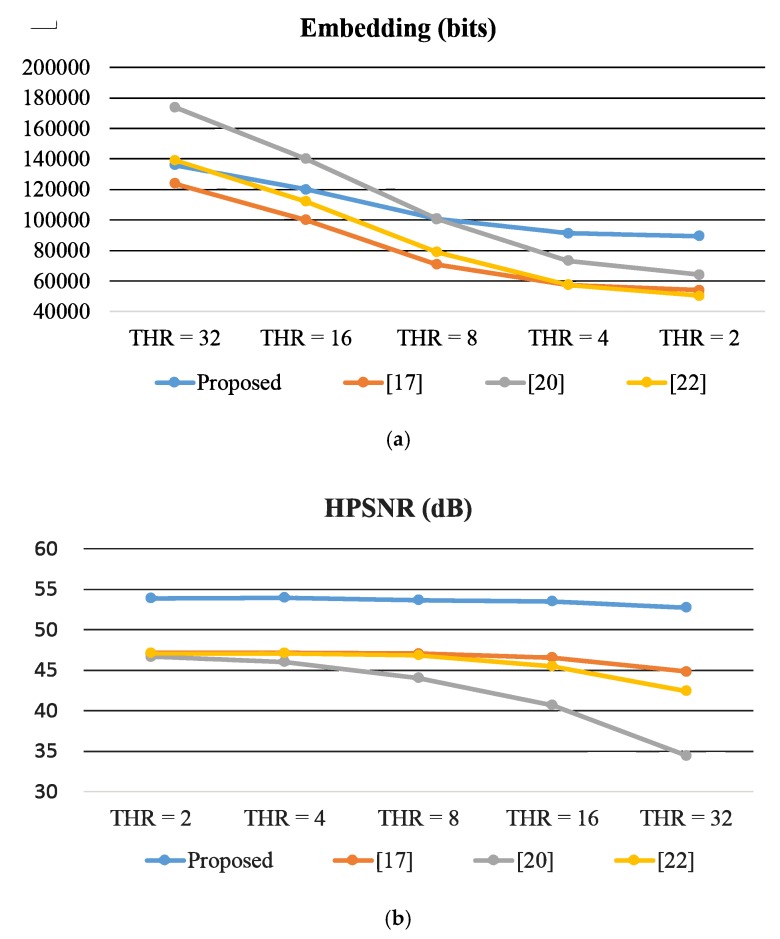
Comparison of the variation of the usage of different thresholds. (**a**) Results of data capacity. (**b**) Results of Human Vision System-based Peak Signal-to-Noise Ratio (HPSNR) value.

Figure 7 presents a comparison of the variation of the usage of the thresholds of 2, 4, 8, 16, and 32 (using the test image Knee as an example). Because the pixel values in a smooth block are close to each other, the data capacity of a smooth block normally higher than that of a complex block. Thus, for all the methods, when the threshold is larger, more blocks are classified as smooth blocks, and larger average embedding capacity is yielded (Figure 6a). However, the quality of the stego image deteriorates accordingly (Figure 6b). The capacity of a pair of blocks in the proposed scheme is 12 bits, which is larger than the other three methods. Therefore, as the threshold increased from 32 to 2, capacity in the proposed scheme does not considerably decrease. The method in [20] exhibits maximum capacity when thresholds are 16 and 32 because in [20], the capacity of a complex block is given by $1+\log_{2}Thr$, where Thr indicates the threshold value. However, the image quality (HPSNR) of [20] abruptly decreases from Thr=8 to Thr=32. In contrast, in the proposed method, the image quality is maintained to a certain extent.

**Figure 8:**
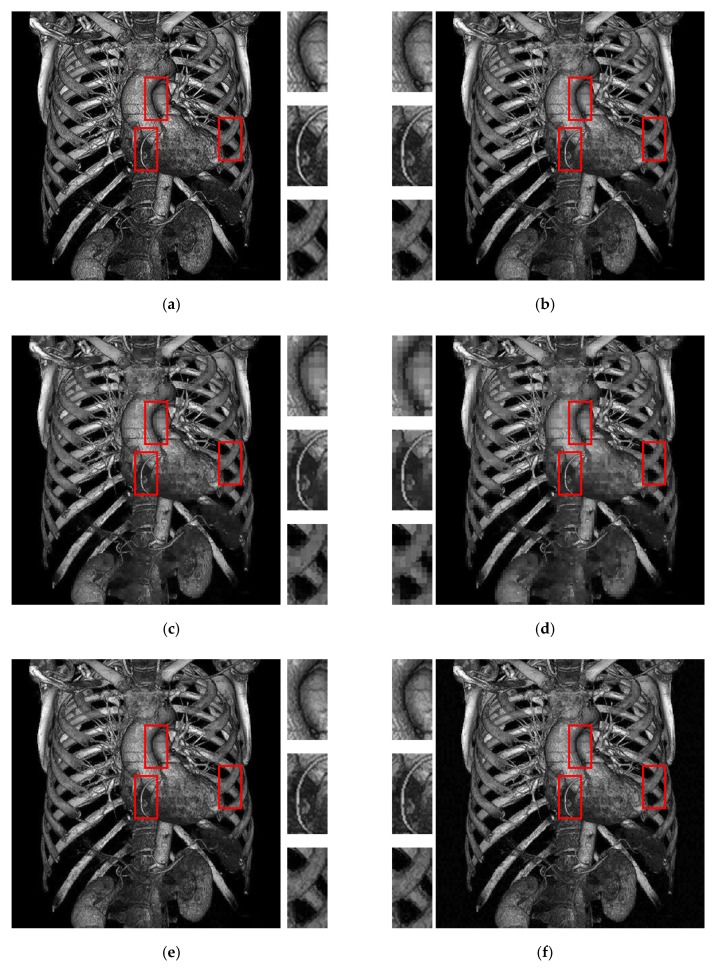
Results of visual comparison using the test image Artifix. (**a**) Original grayscale. (**b**) Pure AMBTC without data embedding. (**c**) Result of [17]. (**d**) Result of [20]. (**e**) Result of [22]. (**f**) Result of the proposed method. From (**c**) to (**f**), the threshold value is set as 8.

Figure 8 shows the visual comparison of different methods. In Figure 8, the threshold is set as 8 for all methods. Compared with the other methods, the proposed method exhibits clearer results in the central parts, and the color is closer to the original grayscale image. The rib area (the right red box) in Figure 8f shows that the proposed method preserves the required details of the original image. For the methods in [17,20,22], the presence of block effects is observed, particularly in Figure 8c,d because the data embedding is based on each AMBTC block. By contrast, in the proposed method, quality improvement phase successfully alleviates these unnatural effects.

**Figure 9:**
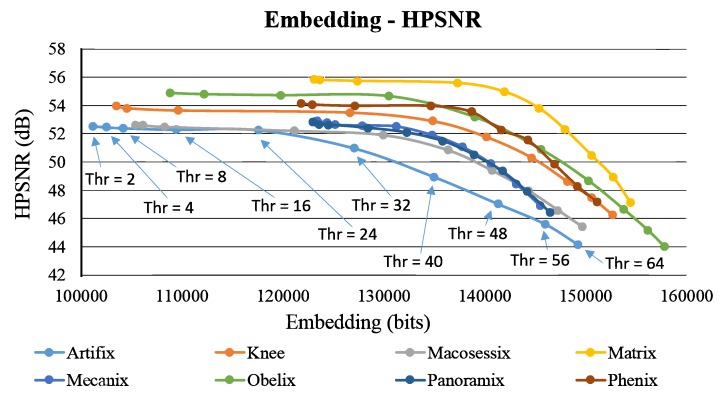
Results of self-evaluation using different threshold values in terms of embedding capacity versus HPSNR.

**Figure 10:**
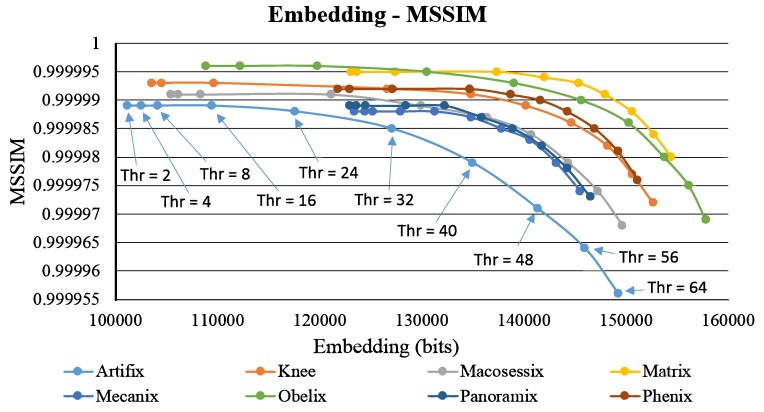
Results of self-evaluation using different threshold values in terms of embedding capacity versus Mean Structural Similarly Index Measure (MSSIM).

**Figure 11:**
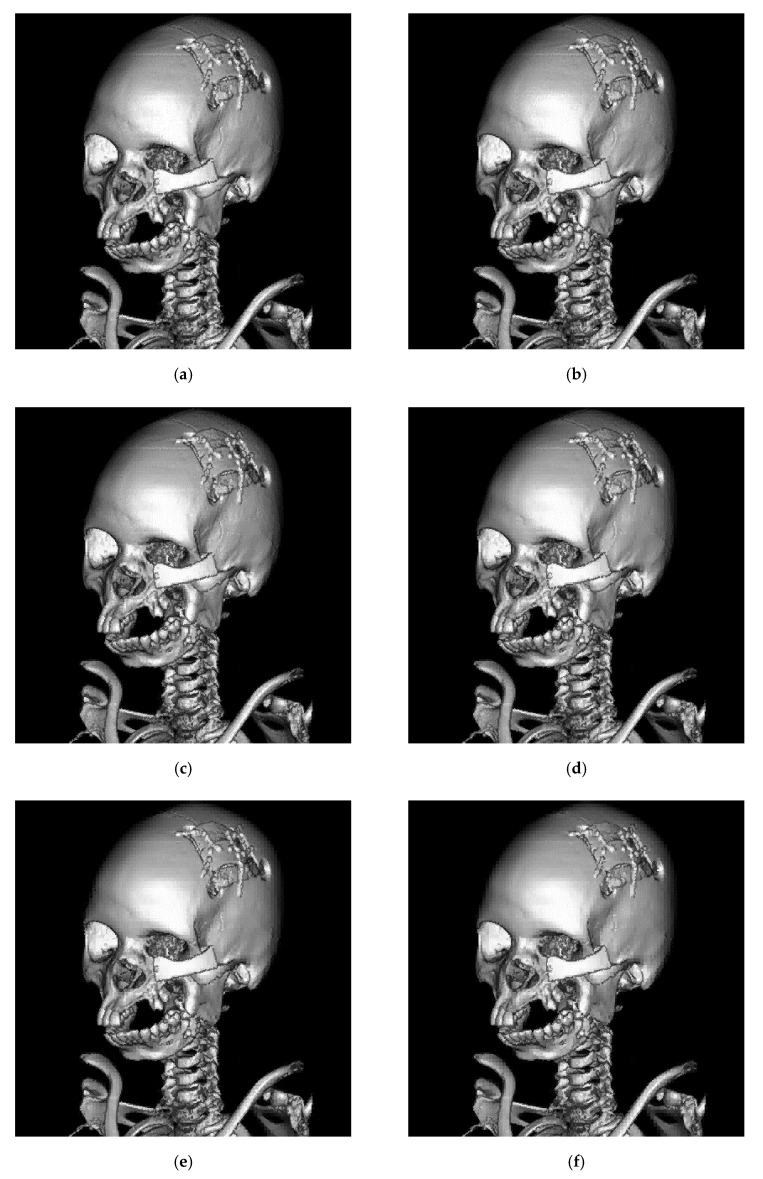
Results of the proposed method using the test image Phenix (with different threshold values). (**a**) Threshold = 2. (**b**) Threshold = 4. (**c**) Threshold = 8. (**d**) Threshold = 16. (**e**) Threshold = 24. (**f**) Threshold = 32. It can be seen in (**e**) and (**f**) that some artifacts occur in the hindbrain region.

Figure 9 and Figure 10 show the self-evaluation results. Similar to Figure 7, the plot of embedding capacity versus image quality is analyzed. When the threshold is higher than 32, the overall capacity abruptly increases, and a considerable amount of secret data are embedded, which deteriorates image quality. Therefore, in both figures, the image quality curves obviously drop in the right side. However, for these eight test images, with the increasing capacity (under a larger threshold), the reduction in image quality is observed from Thr=2 to Thr=16. We find that the declination in the curve is not evident within this range. This phenomenon occurs particularly in the images with more flat regions, such as Matrix, Panoramix, and Phenix. Figure 11 shows the results of the proposed method using the test image Phenix (with different threshold values).

**Figure 12:**
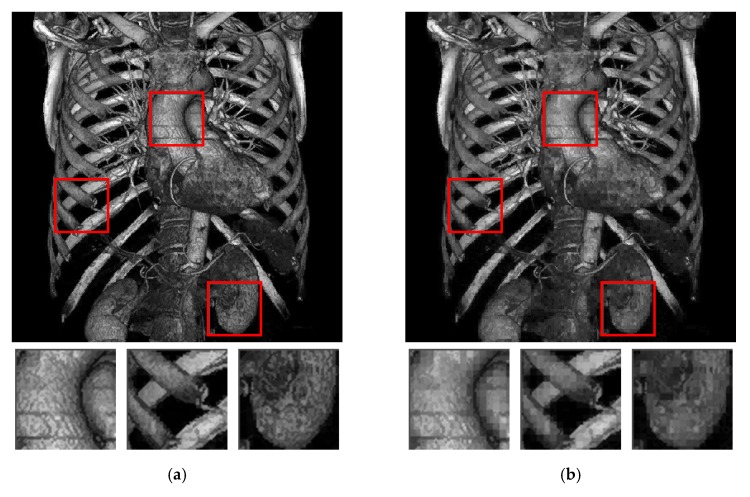
The difference of whether using the inter-block embedding scheme. (**a**) Result of using both intra- and inter-block embedding schemes. (**b**) Result of using only the intra-block embedding scheme.

Finally, we compare the difference of whether using the inter-block embedding scheme. Figure 12 shows that both stego images have the same capacity with an average capacity of 6 bits per block. Figure 12a presents the application of both intra- and inter-block embedding schemes simultaneously, whereas Figure 12b shows the application of only the intra-block embedding scheme. In Figure 12a, 5-bit secret data are embedded by forcing the hidden function value to be one of 32 possibilities, and the other secret bit is embedded through reversible inter-block embedding. Figure 12b shows the embedding of 6-bit secret data embedded into one block. Thus, the hidden function value in Figure 12b becomes one of 64 possibilities (according to the uncontrolled and random secret data), which severely changes AMBTC parameters and causes image degradation. Although the image quality improvement phase is applied in both figures, the result shown in Figure 12a apparently demonstrates a more pleasing visual quality.

## 5. Conclusions

In the era of network and IoT, data hiding becomes a critical research topic for increasing the security of image data transmitted among IoTs. In the past, “seeing is believing” may have been a disputable claim. Today, however, tampering or counterfeiting digital images using current technologies presents no difficulty. It raises a challenge of IoT security and, might, even obstruct the progress of the popularization of IoT. To address this problem, this study presents a data hiding method using the intra- and inter-block embedding strategy. By embedding additional verification code or security-related data in image signal transmission, data hiding technique is an effective solution to the security problem. Embedding data while maintaining good image quality is difficult because the hidden data are random and uncontrollable. Therefore, this work integrates the modified DBS optimization framework into data hiding. As it can be seen in Table 3 and Table 4, the proposed method can embed averagely 121,792 (and 128,712) bits with the average HPSNR values 54.594 dB (and 53.989 dB), which validates the superiority of the proposed method. Compared with the state-of-the-art methods, the proposed method achieves better performance in terms of payload, HPSNR and MSSIM. In our next step, an extended framework about color image steganography is considered.

## Figures and Tables

**Table 1:** Rule of adjusting hidden function value by nominal parameters.

Form of the Trio Code	Change of the Nominal Parameters	Change of the Hidden Function Value
$\left(H_{i},L_{i},B_{i}\right)$	NH, NL, BS	H(θ)
$\left(L_{i},H_{i},{\bar{B}}_{i}\right)$	$\begin{array}{l} NH^{\prime }=NH+1,\;\\ NL^{\prime }=NL+2,\;\\ BS^{\prime }=BS+1 \end{array}$	$H(\theta^{\prime })=H(\theta )+16$

**Table 2:** Rule of inter-block embedding.

2-Bit Secret Code	Change of the Mean Value Difference (*DH*, *DL*)
code 00	(DH, DL) is maintained
code 01	(DH, DL+1) as $LM_{e}^{\prime \prime }=LM_{e}+1$
code 10	(DH+1, DL) as $HM_{e}^{\prime \prime }=HM_{e}+1$
code 11	(DH+1, DL+1) as $HM_{e}^{\prime \prime }=HM_{e}+1$ and $LM_{e}^{\prime \prime }=LM_{e}+1$

**Table 3:** Overall comparison of the four methods (when the threshold value is 2).

Methods	Metrics	Test Images							
		Figure 6a	Figure 6b	Figure 6c	Figure 6d	Figure 6e	Figure 6f	Figure 6g	Figure 6h
AMBTC	HPSNR	52.386	53.714	52.327	56.880	51.947	55.782	51.997	53.398
	MSSIM	0.999989	0.999993	0.999991	0.999998	0.999988	0.999996	0.999989	0.999992
[17]	Payload	47140	54040	53500	113620	90940	134800	86230	86755
	HPSNR	45.627	47.150	46.046	50.836	45.050	48.919	45.290	46.766
	MSSIM	0.985399	0.989024	0.991863	0.996137	0.991472	0.997172	0.992645	0.992822
[20]	Payload	56285	64040	62915	119825	100625	144560	95705	95885
	HPSNR	45.305	46.676	45.621	49.429	44.617	47.689	44.828	46.192
	MSSIM	0.983738	0.986889	0.990008	0.990257	0.986442	0.98905	0.988244	0.988436
[22]	Payload	47140	50404	53500	113560	90940	134800	86230	86695
	HPSNR	45.619	47.085	46.040	50.655	45.044	48.920	45.287	46.753
	MSSIM	0.985352	0.988626	0.99183	0.995239	0.991359	0.997161	0.992628	0.992788
Ours	Payload	101136	89360	105416	133600	123408	147720	122944	121792
	HPSNR	52.848	53.871	52.551	57.262	53.136	56.705	53.175	54.594
	MSSIM	0.999989	0.999993	0.999991	0.999998	0.999988	0.999996	0.999989	0.999992

**Table 4:** Overall comparison of the four methods (when the threshold value is 8).

Methods	Metrics	Test Images							
		Figure 6a	Figure 6b	Figure 6c	Figure 6d	Figure 6e	Figure 6f	Figure 6g	Figure 6h
AMBTC	HPSNR	52.386	53.714	52.327	56.880	51.947	55.782	51.997	53.398
	MSSIM	0.999989	0.999993	0.999991	0.999998	0.999988	0.999996	0.999989	0.999992
[17]	Payload	55705	70735	65290	139045	94285	138550	93385	99745
	HPSNR	45.611	47.074	46.000	50.547	45.043	48.918	45.278	46.718
	MSSIM	0.985154	0.987358	0.99123	0.991295	0.991393	0.997105	0.992532	0.991948
[20]	Payload	85225	100375	94090	168190	124075	168355	104503	111643
	HPSNR	43.593	44.052	43.417	43.274	41.751	41.757	45.095	46.517
	MSSIM	0.944949	0.93087	0.944172	0.831976	0.840213	0.748125	0.992271	0.991502
[22]	Payload	61799	78833	72662	156251	105523	155690	90355	93070
	HPSNR	45.451	46.861	45.758	49.688	44.980	48.722	45.223	46.670
	MSSIM	0.984876	0.986678	0.990819	0.988496	0.991152	0.996853	0.992462	0.992346
Ours	Payload	104152	100490	108296	148264	125192	148664	124464	128712
	HPSNR	52.402	53.653	52.489	56.008	52.777	56.152	52.644	53.989
	MSSIM	0.999990	0.999994	0.999992	0.999998	0.999989	0.999996	0.999990	0.999993
